# Decomposed Collaborative Modeling Approach for Probabilistic Fatigue Life Evaluation of Turbine Rotor

**DOI:** 10.3390/ma13143239

**Published:** 2020-07-21

**Authors:** Ying Huang, Guang-Chen Bai, Lu-Kai Song, Bo-Wei Wang

**Affiliations:** 1 School of Energy and Power Engineering, Beihang University, Beijing 100191, China; bhhuangying@buaa.edu.cn (Y.H.); dlxbgc@buaa.edu.cn (G.-C.B.); 2 School of Computer Science and Engineering, Beihang University, Beijing 100191, China; by1506148@buaa.edu.cn

**Keywords:** probabilistic evaluation, low-cycle fatigue, turbine rotor, Kriging model, intelligent algorithm

## Abstract

To improve simulation accuracy and efficiency of probabilistic fatigue life evaluation for turbine rotor, a decomposed collaborative modeling approach is presented. In this approach, the intelligent Kriging modeling (IKM) is firstly proposed by combining the Kriging model (KM) and an intelligent algorithm (named as dynamic multi-island genetic algorithm), to tackle the multi-modality issues for obtaining optimal Kriging parameters. Then, the decomposed collaborative IKM (DCIKM) comes up by fusing the IKM into decomposed collaborative (DC) strategy, to address the high-nonlinearity problems for accelerating simulation efficiency. Moreover, the DCIKM-based probabilistic fatigue life evaluation theory is introduced. The probabilistic fatigue life evaluation of turbine rotor is regarded as case study to verify the presented approach; the evaluation results reveal that the probabilistic fatigue life of turbine rotor is 3296 cycles. The plastic strain range ∆*ε*_p_ and fatigue strength coefficient *σ*_f_′ are the main affecting factors to fatigue life, whose effect probability are 28% and 22%, respectively. By comparing with direct Monte Carlo method, KM method, IKM method and DC response surface method, the presented DCIKM is validated to hold high efficiency and accuracy in probabilistic fatigue life evaluation.

## 1. Introduction

As a hot-end core component of aeroengine, a turbine rotor operates in severe loading environment at a high temperature, high pressure and high speed. These loads often present complex alterability and cyclicity. In this case, the turbine rotor is prone to generate complex plastic deformation, which inevitably leads to low-cycle fatigue failure. As a result, low-cycle fatigue failure becomes the main failure mode of turbine rotor and seriously affects the reliability and security of aeroengine [1,2,3]. To meet the reliability and high-performance requirements, the fatigue life evaluation of turbine rotor is increasingly important in aeroengine design. Moreover, due to the material deviations, load fluctuations and model variabilities, the fatigue life of turbine rotor usually exhibits great dispersion in essence [4,5,6]. Therefore, effective probabilistic fatigue life evaluation of turbine rotor is urgently required to describe these uncertainties and evaluate its reliability. Under these circumstances, probabilistic analysis techniques have emerged to tackle with the multiple points of uncertainty [7,8]. As one important analysis approach, Monte Carlo (MC) simulation holds high computing accuracy and has been widely applied in reliability evaluation and probabilistic design [9,10]. However, due to the excess simulation calculations and unaffordable large-scale tasks, MC simulation holds unacceptably low computing efficiency. To reduce the computing burdens and improve simulation accuracy, surrogate model methods are developed by replacing the time-consuming simulation with surrogate function/model. Classical surrogate models include response surface model (RSM) [11,12], support vector machine (SVM) [13,14] and artificial neural network (ANN) [15,16], etc. Among them, as an exact interpolation tool, Kriging model holds good approximation ability and nonlinear regression ability [17,18,19], and thereby possesses the potential to perform the structural probabilistic analysis. Unfortunately, for complex fatigue probabilistic analysis involving high complexity and strong nonlinearity, the Kriging model is still hard to establish an accurate mathematical model, because the Kriging optimal algorithm often falls into local optimal Kriging parameters [20,21]. Moreover, for complex multi-layer and multi-response issues (probabilistic fatigue life evaluation of turbine rotor) with strong nonlinearity, the Kriging model is insufficient to describe complex structural responses, resulting in unsatisfactory efficiency and accuracy.

To deal with the multi-modality issues in Kriging parameters searching, we propose an intelligent algorithm to avoid the local optimum and find the global optimal Kriging parameters. As a valuable intelligent algorithm, multi-island genetic algorithm (MIGA) with unique migration operation and parallel computing can address complex optimization problems with prominent searching performance [22,23]. However, due to the fixed crossover rate and mutation rate that is employed, the traditional MIGA is not enough to ensure the population diversity in objective searching [24,25], which would lead to more iterative time and insufficient optimization efficiency. In this paper, to enhance the algorithm performance of MIGA, we further propose a dynamic multi-island genetic algorithm (DMIGA) by designing the dynamic crossover rate and dynamic mutation rate. The DMIGA algorithm is employed to search the optimal Kriging parameters in Kriging modeling process, and then the intelligent Kriging modeling (IKM) shall be obtained. The DMIGA avoids the local optimum and immature convergence defects in multi-modality Kriging parameters problems, which guarantees the modeling efficiency and accuracy of IKM effectively.

To further improve the simulation efficiency in probabilistic fatigue life evaluation, decomposed collaborative (DC) strategy is employed to address the multi-layer and multi-response problems [12,26,27,28]. The DC strategy was firstly proposed to deal with multi-objective multi-disciplinary problems [29], and the effectiveness of DC was verified in radial running clearance control, multi-failure probability design and turbine blade damage analysis [30,31,32]. In these investigations, DC strategy was demonstrated to be a feasible and effective analysis strategy in complex interlayer evaluation problems. However, for turbine rotor of aeroengine, plastic strain range induced by load fluctuations further increases the nonlinearity degree of probabilistic fatigue life evaluation. The unacceptable accuracy and efficiency problems would occur if directly adopting DC strategy with regular surrogate model (such as decomposed collaborative RSM, DCRSM). In this situation, considering the potentials of intelligent Kriging model, we further developed a decomposed collaborative intelligent Kriging model (denoted as DCIKM) to improve the simulation accuracy and efficiency for probabilistic fatigue life evaluation of a turbine rotor.

The objective of this paper is to ameliorate the computational efficiency and accuracy of turbine rotor probabilistic fatigue life evaluation by constructing an efficient and accurate surrogate model (DCIKM). Given the characteristics of fatigue life calculation for turbine rotor, this paper employs intelligent algorithm to optimize Kriging parameters, and combines DC strategy to further decrease the nonlinearity of surrogate modeling. The innovation of this paper lies in that: for assessing the probabilistic fatigue life of turbine rotor considering the plasticity of materials, the corresponding efficient methods are firstly proposed. The proposed DCIKM is verified by probabilistic fatigue life evaluation of aeroengine turbine rotor.

The structure of this paper is arranged as follows. Section 2 discusses the decomposed collaborative modeling approach, including the basic principles and mathematical models of Kriging modeling, intelligent Kriging modeling (IKM) and DCIKM. Section 3 introduces the DCIKM-based probabilistic fatigue life evaluation theory. In Section 4, the probabilistic fatigue life evaluation of turbine rotor is performed to verify the effectiveness of the proposed DCIKM. Some conclusions and outlooks are summarized in Section 5.

## 2. Decomposed Collaborative Modeling Approach

### 2.1. Intelligent Kriging Modeling

#### 2.1.1. Kriging Model Overview

Assuming that the approximated values contains spatial correlation, the Kriging model can acquire the interpolation results by weighting the known values [33,34]. For a m-dimensional input, points *X* = (*X*_1_, *X*_2_, …, *X*_m_)^T^ and their output responses *Y* = (*y*_1_, *y*_2_, …, *y*_m_)^T^, the nonlinear relationship between input points and output responses can be mapped by an interpolation Kriging model, i.e.,

(1) $$Y=\left\{\begin{array}{c} y_{1}\\ \vdots \\ y_{m} \end{array}\right\}=\left[\begin{array}{ccc} f_{1}(X_{1}) & \cdots & f_{p}(X_{1})\\ \vdots & \ddots & \vdots \\ f_{1}(X_{m}) & \cdots & f_{p}(X_{m}) \end{array}\right]\left[\begin{array}{c} \beta_{1}\\ \vdots \\ \beta_{p} \end{array}\right]+\left[\begin{array}{c} \varepsilon_{1}\\ \vdots \\ \varepsilon_{m} \end{array}\right]=F\beta +\varepsilon$$

where *Fβ* represents the regression model, which is employed to approximate the entire design space; *ε* the stochastic process, to describe the local deviation of regression model; Therein, *ε* holds the following statistical characteristics:

(2) $$\varepsilon_{i}∼N(0,\sigma_{\varepsilon }^{2})$$

(3) $$\mathrm{cov}(\varepsilon_{i},\varepsilon_{j})=\sigma_{\varepsilon }^{2}R(\theta ,X_{i},X_{j})$$

where *σ_ε_*^2^ is the process variance, *R*(∙) the spatial correlation function, *θ=*(*θ*_1_, *θ*_2_, …, *θ_n_*) the correlation parameter vector.

In view of the powerful mapping ability in fitting nonlinear limit state functions [20,35], the Gaussian function is regarded as the correlation function in Kriging modeling, i.e.,

(4) $$R(\theta ,X_{i},X_{j})=\prod_{k=1}^{n}\exp[-\theta_{k}{\left(x_{ik}-x_{jk}\right)}^{2}]$$

Therefore, for a given random point *X*_d_, its response value *Ŷ*(*X*_d_) can be estimated by product of weight *w* and known response *Y*, that is *Ŷ*(*X*_d_) = *w*^T^*Y*. The prediction error can be obtained as:

(5) $$\psi (X)=E[{\left(\hat{Y}(X_{d})-Y(X_{d})\right)}^{2}]=\sigma_{\varepsilon }^{2}(1+w^{T}Rw-2w^{T}r)$$

in which

(6) $$r(X)={\left\{R(\theta ,X_{1},X),R(\theta ,X_{2},X),\ldots ,R(\theta ,X_{m},X)\right\}}^{T}$$

(7) $$R=\left(\begin{array}{ccc} R(\theta ,X_{1},X_{1}) & \ldots & R(\theta ,X_{1},X_{m})\\ \vdots & \ddots & \vdots \\ R(\theta ,X_{m},X_{1}) & \cdots & R(\theta ,X_{m},X_{m}) \end{array}\right)$$

where *ψ*(*X*) is the prediction error, *r*(∙) the correlation vector between the given point *X*_d_ and sampling points, ***R***(∙) the correlation matrix.

By minimizing the prediction error *ψ*(*X*), the estimated regression coefficient $\hat{\beta }$ and process variance ${\hat{\sigma }}_{\varepsilon }^{2}$ are expressed as:

(8) $$\begin{array}{l} \hat{\beta }={\left(F^{T}R^{-1}F\right)}^{-1}F^{T}RY\\ {\hat{\sigma }}_{\varepsilon }^{2}=\frac{1}{m}(Y-F\hat{\beta })R^{-1}(Y-F\hat{\beta }) \end{array}$$

From Equations (7)–(8), the regression coefficient $\hat{\beta }$ and process variance ${\hat{\sigma }}_{\varepsilon }^{2}$ heavily depend on the correlation parameter *θ*, which can be obtained by the maximum likelihood estimation method [36].

(9) $$\min\left\{{\left|R\right|}^{1/m}{\hat{\sigma }}_{\varepsilon }^{2}\right\}$$

Once the optimal correlation parameters θ* are acquired, the Kriging model and the corresponding prediction error are acquired:

(10) $$\hat{Y}(X)=f^{T}(X)\hat{\beta }+r^{T}(X)R^{-1}(Y-F\hat{\beta })$$

(11) $$\begin{array}{l} MSE\left\{\hat{Y}(X)\right\}={\hat{\sigma }}_{\varepsilon }^{2}\left\{1+u^{T}(X)[{\left(F^{T}R^{-1}F\right)}^{-1}u(X)-r^{T}R^{-1}r(X)]\right\}\\ \;s.t.\;u(X)=F^{T}R^{-1}r(X)-f(X) \end{array}$$

where *MSE*{∙} means the mean square error function. Obviously, the Kriging interpolation precision and model performance heavily depend on the correlation parameters *θ*. Once the optimal correlation parameters θ* are searched, the accurate Kriging model will be obtained correspondingly. Therefore, the optimization problems of correlation parameters should be regarded as the main focus of surrogate modeling problems.

#### 2.1.2. Intelligent Algorithm

**Figure 1:**
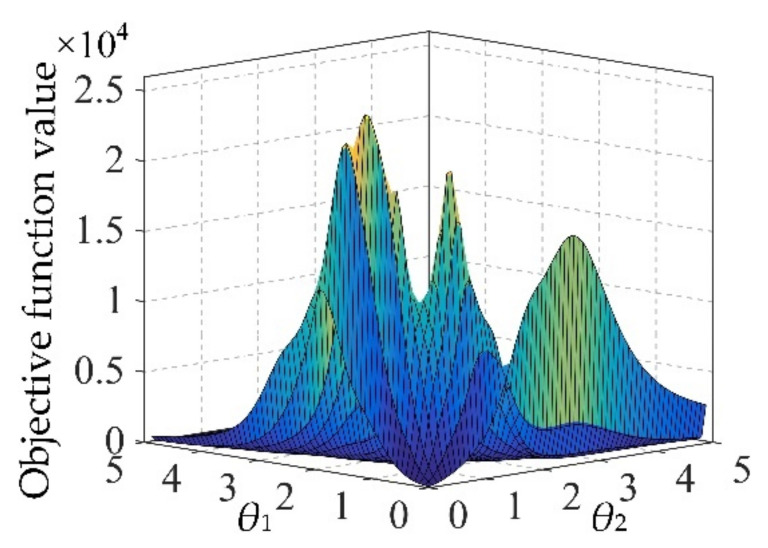
Multi-modality characteristics in Kriging modeling.

**Figure 2:**
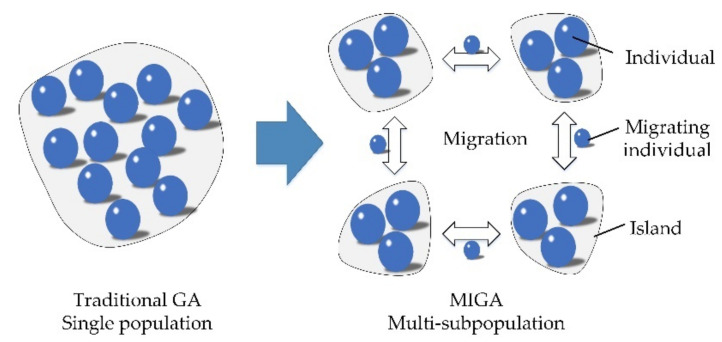
Migration operation of dynamic multi-island genetic algorithm (DMIGA).

In traditional parameters optimization of Kriging modeling, the generalized pattern optimal algorithm is often used in the sense of maximum likelihood [37]. The Kriging parameters optimization problem is shown in Equation (12). However, owing to the sensitive dependence on initial point, this pattern optimal algorithm often falls into local optimum and resulting in unacceptable prediction error. Moreover, the objective function in Kriging modeling shows complex multi-modality characteristics (shown in Figure 1) in probabilistic fatigue life evaluation, which increases the difficulty to find the global optimum. In this case, to avoid the complex gradient calculations and guarantee the optimization effect, an intelligent algorithm called the dynamic multi-island genetic algorithm (DMIGA) is proposed. DMIGA generates the potential optimums as individuals in several islands (or populations), and then chooses the best-performance individuals to evolve into the next iteration. As the individuals evolving and migrating between multi-islands [23], population diversity is ensured, and the global optimum is finally acquired. The migration operation of DMIGA is drawn in Figure 2.

(12) $$\left\{\begin{array}{ll} \mathrm{find} & \theta =(\theta_{1},\theta_{2},\ldots ,\theta_{n})\\ \min & f(\theta )={\left|R\right|}^{1/m}{\hat{\sigma }}_{\varepsilon }^{2}\\ s.t. & \theta_{i}>0,\;i=1,2,\ldots ,n \end{array}\right.$$

where *θ_i_* is the *i*-th correlation parameters; *n* the number of input variables; *f*(*θ*) the fitness function.

To ensure the species diversity of each island in DMIGA, dynamic crossover rate and dynamic mutation rate are proposed in this study: when the dispersion degree arcsin(*f*_ave_/*f*_max_) is greater than some certain value, the high-quality individuals are difficult to generate due to the lack of the species diversity, then the dynamic mutation operation would be set with a high mutation rate to enhance the mutation rate; otherwise the dynamic crossover operation would be set with a high crossover rate to enhance the crossover rate [38]. The arcsin(*f*_ave_/*f*_max_) is adopted as the judgment function of species diversity, since it can change faster with the increase of average fitness *f*_ave_. The dynamic crossover rate *p*_c_ and dynamic mutation rate *p*_m_ are designed as

(13) $$p_{c}=\left\{\begin{array}{ll} k_{1}\frac{\arcsin(f_{\mathrm{ave}}/f_{\max})}{\pi /2} & \arcsin(f_{\mathrm{ave}}/f_{\max})<\pi /6\\ k_{1}\left[1-\frac{\arcsin(f_{\mathrm{ave}}/f_{\max})}{\pi /2}\right] & \arcsin(f_{\mathrm{ave}}/f_{\max})\geq \pi /6 \end{array}\right.$$

(14) $$p_{m}=\left\{\begin{array}{ll} k_{2}\left[1-\frac{\arcsin(f_{\mathrm{ave}}/f_{\max})}{\pi /2}\right] & \mathrm{arcisn}(f_{\mathrm{ave}}/f_{\max})<\pi /6\\ k_{2}\frac{\arcsin(f_{\mathrm{ave}}/f_{\max})}{\pi /2} & \arcsin(f_{\mathrm{ave}}/f_{\max})\geq \pi /6 \end{array}\right.$$

where *f*_ave_ indicates average fitness; *f*_max_ the maximum fitness; arcsin(∙) the arc sine function. In this paper, *k*_1_ and *k*_2_ are chosen as 1 and 0.005, respectively. Moreover, π/6 is adopted as a guideline because arcsin(*f*_ave_/*f*_max_) ≥ π/ 6 equals to *f*_ave_/*f*_max_ ≥ 1/2, which reflects the diversity of species. Furthermore, the reason for dividing by π/2 is to ensure that arcsin(*f*_ave_/*f*_max_)/(π/2) ≤ 1.

**Figure 3:**
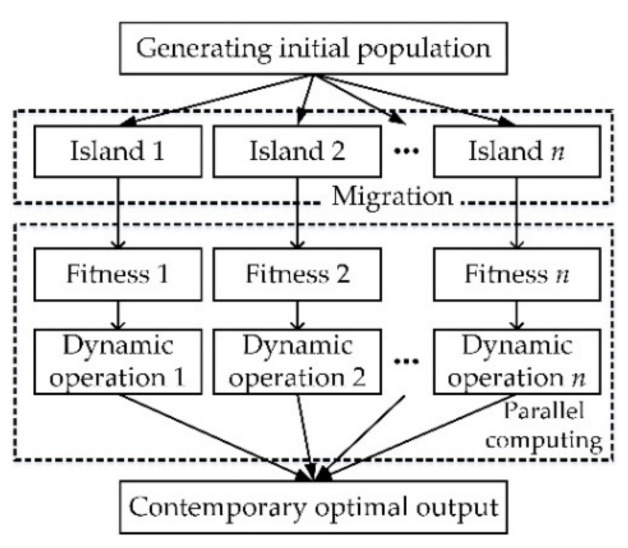
θ* searching procedure with DMIGA.

With the DMIGA, the optimal Kriging parameters θ* shall be searched as follows: Firstly, an initial population is generated and divided into several subpopulations, with each individual in subpopulation is a potential optimal θ*. Then the fitness values (i.e., Equation (12)) of all individuals are calculated in each subpopulation and the dynamic crossover operation and dynamic mutation operation are performed correspondingly. Finally, the global optimum is obtained by running multiprocessors simultaneously. The θ* searching procedure with DMIGA is shown in Figure 3.

#### 2.1.3. Intelligent Algorithm with Kriging Model, IKM

To ameliorate the computational accuracy of probabilistic fatigue life evaluation, an intelligent Kriging model (IKM) is proposed based on Kriging model with nonlinear mapping ability and DMIGA with efficient global search ability. The modeling thought of IKM and the corresponding probabilistic analysis principle is summarized as follows:

Firstly, the finite element (FE) model of turbine rotor is established and the thermal-structure FE analysis considering material plasticity is performed to gain training and testing data.

Secondly, in view of the sample data, the regression coefficients $\hat{\beta }$ and process variance ${\hat{\sigma }}_{\varepsilon }^{2}$ are obtained, the expressions of fitness function (Equation (12)) is constructed.

Then, by employing DMIGA to solve Equation (12), the optimal correlation parameter θ* is acquired and the IKM is built. The corresponding regression coefficient $\beta^{*}$, process variance ${\hat{\sigma }}_{*}^{2}$ and the prediction result Y*(*X*_p_) are obtained:

(15) $$\left\{\begin{array}{l} \beta^{*}={\left(F^{T}R^{*}{}^{-1}F\right)}^{-1}F^{T}R^{*}Y\\ {\hat{\sigma }}_{*}^{2}=\frac{1}{m}(Y-F\beta^{*})R^{*}{}^{-1}(Y-F\beta^{*}) \end{array}\right.$$

(16) $$Y^{*}(X_{p})=f^{T}(X_{p})\beta^{*}+r^{T}(X_{p})R^{*}{}^{-1}(Y-F\beta^{*})$$

where $\beta^{*}$, ${\hat{\sigma }}_{*}^{2}$ and R* are the regression coefficient, process variance and correlation parameters matrix corresponding to the optimal correlation parameter θ*, respectively.

**Figure 4:**
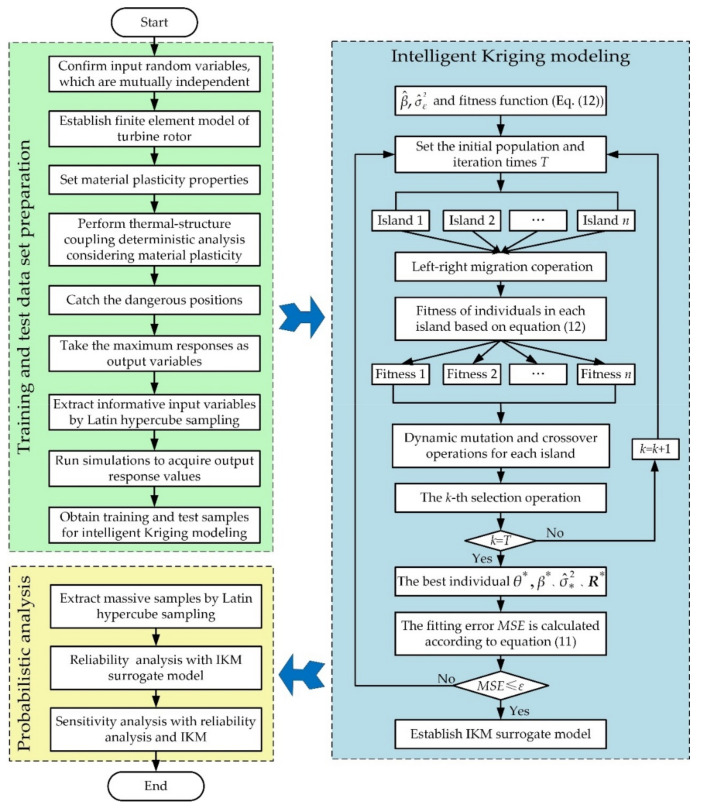
Flow chart of probabilistic analysis with intelligent Kriging modeling (IKM).

Finally, considering the established IKM surrogate model and a large number of sample data, the probabilistic analysis is completed. The flow chart of probabilistic analysis with IKM is shown in Figure 4.

### 2.2. Decomposed Collaborative IKM, DCIKM

#### 2.2.1. Basic thought of DCIKM

Probabilistic fatigue life evaluation of turbine rotor involves multi-layer (FE simulation layer, model prediction layer, etc.) and multi-response (mean stress, elastic strain range and plastic strain range, etc.), which brings high complexity and strong nonlinearity to construct surrogate model. DC strategy is a highly efficient and precise high-precision analysis technique for structural multi-failure and multi-objective reliability design [39,40]. Along with the heuristic thought, the decomposed collaborative IKM (DCIKM) is developed for probabilistic fatigue life evaluation in respect of IKM. The basic idea of DCIKM is summarized as follows:

1. Regarding the evaluation layer and response traits, the complex model with all input variables and total output response is divided into multiple simple submodels, each of which contains fewer input variables and one output response. It is assumed that the submodels are independent of each other.
2. Considering the plasticity of materials, the thermal-structure coupling deterministic analysis is accomplished through FE simulation.
3. The output responses of sub-models are obtained by importing several input variables into FE calculation, and the input variables and output responses are treated as training and testing data.
4. With the extracted samples, the decomposed IKM of sub-models are constructed by the proposed IKM thought.
5. Massive sampling for input variables is performed by Latin hypercube sampling (LHS) technique, and the statistical characteristics of output responses are obtained by decomposed IKM simulation.
6. Taking the output responses of decomposed IKM models as the input variables, the collaborative IKM is established. By employing the simple DCIKM approach instead of time-consuming direct MC simulation, the probabilistic fatigue life evaluation is accomplished.

In light of the above analysis, this analysis process is equivalent to decomposing complex multi-layer analysis into a series of simple decomposed IKM analyses and collaborative IKM analysis. Evidently, with the progressing of decomposition course, more input variables in a complex overall model are decomposed into fewer input variables in simple sub-models, which is conducive to reduce the nonlinearity of fitted function and the coupling effect between variables. Therefore, by combining the nonlinear modeling ability of the proposed IKM model and the simplifying computation ability of DC strategy, the proposed DCIKM possesses the potential to ameliorate efficiency and accuracy for complex probabilistic fatigue life evaluation.

#### 2.2.2. Mathematical Modeling of DCIKM

Assuming that the probabilistic fatigue life evaluation refers to *r* layers, then the complex multi-layer problems are transformed into a series of simple single-layer problems by DC strategy. When *X*^(*p*)^ represents the input variable of the *p*-th layer, the corresponding output response *Y*^(*p*)^ is:

(17) $$\left\{\begin{array}{l} Y^{\left(p\right)}=f(X^{\left(p\right)}),\;p=1,\;2,\cdot \cdot \cdot ,r\\ X^{\left(p\right)}={\left[X_{1}{}^{\left(p\right)},X_{2}{}^{\left(p\right)},\cdots ,X_{n}{}^{\left(p\right)}\right]}^{T} \end{array}\right.$$

The output response *Y*^(*p*)^ of surrogate model at the *p*-layer can be described as:

(18) $$Y^{\left(p\right)}=f(X^{\left(p\right)})=f^{T}(X^{\left(p\right)}){\tilde{\beta }}^{*}+r^{T}(X^{\left(p\right)}){\tilde{R}}^{-1}(\tilde{Y}\;-\tilde{F}{\tilde{\beta }}^{*})$$

Equation (18) is a decomposed IKM model, where ${\tilde{\beta }}^{*}$, $\tilde{R}$, $\tilde{Y}$, $\tilde{F}$ is the calculated regression coefficient, correlation parameter matrix, sample response value, regression function in *p*-th layer, respectively.

Regarding the output responses of all layers {*Y*^(1)^, *Y*^(2)^, …, *Y*^(*r*)^} as input variables $\bar{X}$, $\bar{Y}$ represents the output response of overall surrogate model, then the collaborative IKM model is constructed as:

(19) $$\left\{\begin{array}{l} \bar{Y}=f(\bar{X})=f^{T}(\bar{X}){\bar{\beta }}^{*}+r^{T}(\bar{X}){\bar{R}}^{-1}(\bar{Y}\;-\bar{F}{\bar{\beta }}^{*})\\ \bar{X}={\left[{\bar{X}}_{1},{\bar{X}}_{2},\ldots ,{\bar{X}}_{r}\right]}^{T} \end{array}\right.$$

where ${\bar{\beta }}^{*}$, $\bar{R}$, $\bar{Y}$ and $\bar{F}$ are the predicted regression coefficients, correlation parameters matrix, sample response values, and regression function, respectively.

**Figure 5:**
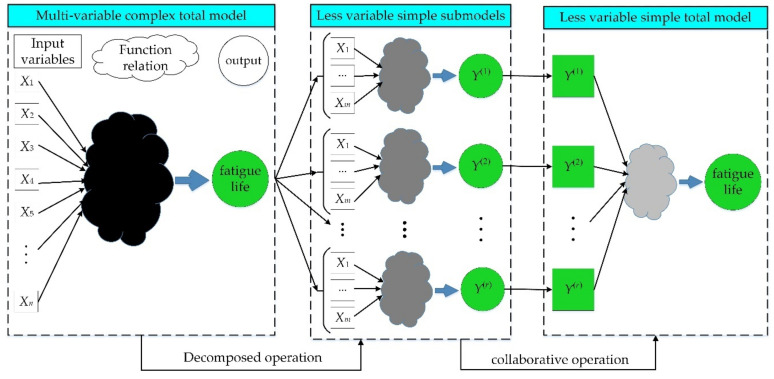
Modeling procedure of the presented decomposed collaborative intelligent Kriging model (DCIKM).

The above decomposed collaborative modeling process can be vividly drawn in Figure 5. Namely, the complex IKM model of overall fatigue life prediction is first divided into decomposed IKM sub-models at different layers (Equation (18)), and then the collaborative IKM (Equation (19)) is achieved to perform the probabilistic calculations. This method is called as decomposed collaborative intelligent Kriging model (DCIKM) method, which is suitable for multi-layer and multi-response probabilistic fatigue life evaluation.

## 3. Probabilistic Fatigue Life Evaluation Theory

Probabilistic analysis, mainly consists of reliability analysis and sensitivity analysis, is to assess system reliability and find the main factors affecting system reliability. As for fatigue life evaluation, probabilistic analysis is to quantify the reliability of system and explore the influence degree of each uncertainty parameter. For the complex multi-layer probabilistic analysis [12,21], a DCIKM-based probabilistic fatigue life evaluation theory is developed to improve the simulation efficiency and accuracy, which is introduced as follows.

Sensitivity reflects the influence level of input random variables to output response, which helps to identify the main affecting factors and guides the structural design. When the acceptable output response of the *p*-th layer is [*Y*^(*p*)^], the limit state function *G*^(*p*)^(*x*) based on decomposed IKM model can be expressed as:

(20) $$G^{\left(p\right)}(x)=\left[Y^{\left(p\right)}\right]-\left[f^{T}(X^{\left(p\right)}){\tilde{\beta }}^{*}+r^{T}(X^{\left(p\right)}){\tilde{R}}^{-1}(\tilde{Y}\;-\;\tilde{F}{\tilde{\beta }}^{*})\right],\;p=1,2,\ldots ,r$$

in which the value of limit state function *G*^(*p*)^(*x*) can be determined by the indicator function of failure domain:

(21) $$I_{f}[G^{\left(p\right)}(x_{l})]=\left\{\begin{array}{ll} 1, & G^{\left(p\right)}(x_{l})\leq 0\\ 0, & G^{\left(p\right)}(x_{l})>0 \end{array}\right.$$

where *x_l_* indicates the *l*-th data set; *l* = 1, 2, …, *s*.

Through a large number of sampling of input variables [41], the sensitivity values of input variables can be obtained by the decomposed IKM:

(22) $$S^{\left(p\right)}=Mean\left(\frac{I_{f}[G^{\left(p\right)}(x_{l})](x_{il}-Mean(x_{i}))}{Var(x_{i})}\right)$$

where *x_i_* means the *i*-th input vector of all sample variables; *Mean*(·) the mean function; *Var*(·) the variance function. 

Assuming that the whole output allowable value is $\left[\bar{Y}\right]$, the limit state function *G*(*x*) based on collaborative IKM can be described as:

(23) $$G(x)=[\bar{Y}]-\left[f^{T}\left(\bar{X}\right){\bar{\beta }}^{*}+r^{T}\left(\bar{X}\;\right){\bar{R}}^{-1}(\bar{Y}-\;\bar{F}{\bar{\beta }}^{*})\right]$$

Through the collaborative IKM model and random sampling method [29], the fatigue reliability is denoted by:

(24) $$\begin{array}{l} I_{r}[G(x_{l})]=\left\{\begin{array}{ll} 1, & G(x_{l})\geq 0\\ 0, & G(x_{l})<0 \end{array}\right.\\ R=\frac{1}{N}\sum_{i=1}^{N}I_{r}[G(x_{l})]=\frac{N_{r}}{N} \end{array}$$

where *I_r_*[*G*(*x_l_*)] is the indicator function of secure domain; *N_r_* the sample number in secure domain; *N* the total sample number.

Moreover, the global failure probability and the sensitivity of input variables to overall output response are calculated as [41]:

(25) $$I_{f}[G(x_{l})]=\left\{\begin{array}{ll} 1, & G(x_{l})\leq 0\\ 0, & G(x_{l})>0 \end{array}\right.$$

(26) $$\left\{\begin{array}{l} P=\frac{1}{N}\sum_{i=1}^{N}I_{f}[G(x_{l})]=\frac{N_{b}}{N}\\ S=Mean\left(\frac{I_{f}[G(x_{l})](x_{il}-Mean(x_{i}))}{Var(x_{i})}\right) \end{array}\right.$$

where, *P* indicates the total failure probability; *S* the sensitivity of input variables to overall output response; *I_f_*[*G*(*x_l_*)] the indicator function of total failure domain; *N*_b_ the sample number of total failure domain.

## 4. Case Study

In this section, the probabilistic fatigue life evaluation of turbine rotor considering the material plasticity is performed. Following the efficiency and accuracy for the proposed DCIKM approach, the reliability-based fatigue life evaluation is completed and the importance degree of input variables are quantified.

### 4.1. Material Preparations

#### 4.1.1. Finite Element Model

**Figure 6:**
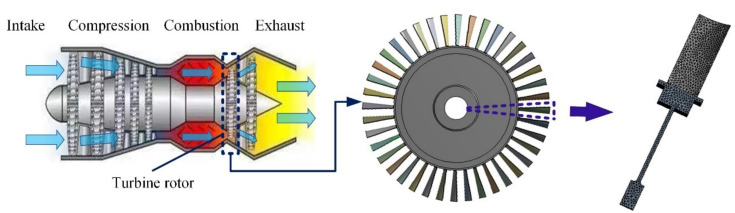
Schematic diagram of aeroengine turbine rotor.

To reduce the computational complexity and task volume, 1/40 of turbine rotor [3] are selected as study object. The schematic diagram of aeroengine turbine rotor is shown in Figure 6. Based on the coarse grid division, we improved the grid quality by setting the “minimum size” as 4.5 × 10^−3^ m and “minimum edge length” as 1.9995 × 10^−3^ m, and refined the local mesh of blade root by setting “element size” as 3.6 × 10^−3^ m. From the convergence effects and simulation accuracy in reference [3,30], with the set meshing procedures, the meshing effects is guaranteed effectively. As shown in Figure 6, the FE model of simplified turbine rotor involves 18,454 quadrilateral elements and 30,911 element nodes. Moreover, an appropriate symmetric boundary constraint is imposed on the sector disc, and axial and circumferential constraints are loaded on the inner diameter arc. Furthermore, for facilitating the fatigue life calculation, we simplify the actual complex load spectrum of turbine rotor into the trapezoidal load spectrum [32]. 

#### 4.1.2. Variable Selection

In this study, the nickel-based superalloy GH4133B was selected as turbine rotor material. Although GH4133B may have creep fatigue failure in high temperature environment, the plastic strain generated in blade root area leads to turbine rotor being more prone to occur low cycle fatigue (LCF) failure. Therefore, this paper mainly considers LCF failure of turbine rotor. Moreover, LCF life of turbine rotor often shows great dispersion affected by multiple uncertainties, hence it is necessary to perform probabilistic LCF life assessment for turbine rotor. To accurately describe the life dispersion and quantify the probabilistic fatigue life of turbine rotor, the physical uncertain parameters of turbine rotor, such as rotor speed *ω*, gas temperature *T*, elastic modulus *E*, material density *ρ*, thermal conductivity *λ*, thermal expansion coefficient *α*, are considered as the first part of input random variables, whose distribution characteristics [21,27,32] are shown in Table 1. Furthermore, the parameters in modified Manson–Coffin [42,43] models, such as fatigue strength exponent *b*, fatigue ductility exponent *c*, fatigue strength coefficient *σ*_f_′ and fatigue ductility coefficient *ε*_f_′, are considered as the second part of input random variables and its distribution characteristics are shown in Table 2.

In view of the values of elastic modulus *E*, heat conductivity *λ*, thermal expansion coefficient *α* are varying with temperature, we introduce its nonlinear variation characteristics in Table 3. Moreover, to fully capture the limit state surface information, the variable distribution characteristics in Table 1 are matched with the aircraft cruise state. Furthermore, the sampling range of input variables is determined as [μ − f_*_σ, *μ* + f_*_σ] (where *μ* is the variable mean, *σ* the standard deviation, *f* the positive constant). The parameter *f* determines the sampling domain range, *f* is set to 4 in this study, since this sampling domain contains 99.99% variable fluctuation information [44] and the corresponding failure possibility.

### 4.2. Deterministic Fatigue Life Evaluation

**Figure 7:**
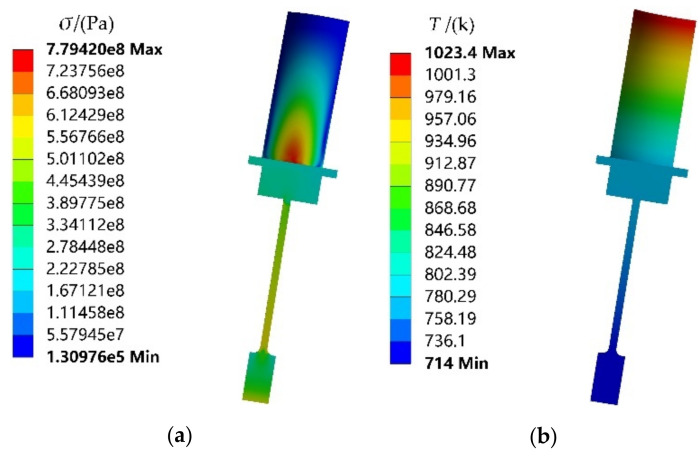

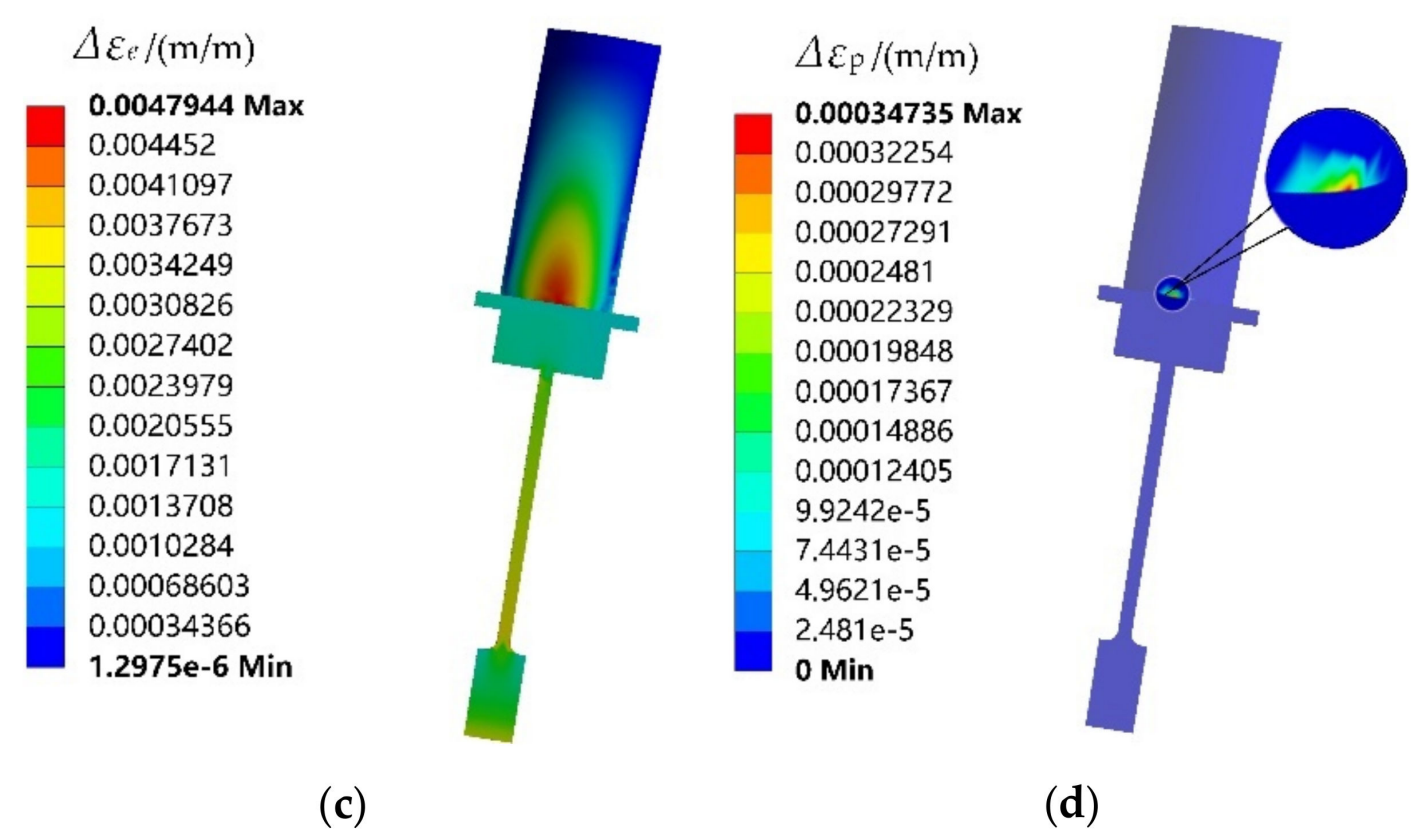
Nephogram of the stress-strain and temperature for turbine rotor: (**a**) stress, (**b**) temperature, (**c**) elastic strain range, (**d**) plastic strain range.

By importing the mean values of material properties and load parameters into FE model, thermal-structure coupling analysis is performed, the maximum stress and the temperature distribution of turbine rotor are acquired in Figure 7a,b, respectively. From the stress and temperature distribution, we find that the back section in blade root is the critical point and its stress and temperature are 779.4 MPa and 778.35 K, respectively. Since the turbine rotor does not generate compressive stress, then the negative half axis in hysteresis loop does not occur. Therefore, we regard the residual strain as plastic strain range, and the difference value between total strain and plastic strain range as elastic strain range. The elastic strain and plastic strain distribution are shown in Figure 7c,d. To clearly exhibit the distribution traits of plastic strain range, we further enlarge the plastic strain area. which is drawn in Figure 7d. Note that the material occurs plastic strain in critical point as shown in Figure 7d, which needs to be considered in fatigue life evaluation. Considering the maximum stress cycle 0-*σ*_max_-0, the mean stress *σ*_m_ is obtained as 0.5 × (*σ*_min_+*σ*_max_). With the calculated mean stress *σ*_m_, elastic strain range ∆*ε*_e_, plastic strain range ∆*ε*_p_ and Manson–Coffin model, the low-cycle fatigue life of turbine rotor is obtained as 3606 cycles. Manson–Coffin model is shown in Equation (27).

(27) $$\frac{\Delta \varepsilon }{2}=\frac{\Delta \varepsilon_{e}+\Delta \varepsilon_{p}}{2}=\frac{{\sigma^{\prime }}_{f}-\sigma_{m}}{E}{\left(2N_{f}\right)}^{b}+{\varepsilon^{\prime }}_{f}{\left(2N_{f}\right)}^{c}$$

where ∆*ε* represents the total strain range; *E* the modulus of elasticity; *σ*_f_′ the fatigue strength coefficient; *ε_f_′* the fatigue ductility coefficient; *b* the fatigue strength index; *c* the fatigue ductility index; and *N*_f_ the failure cycle number.

### 4.3. Decomposed Stress-Strain Prediction

#### 4.3.1. Decomposed IKM Modeling

**Figure 8:**
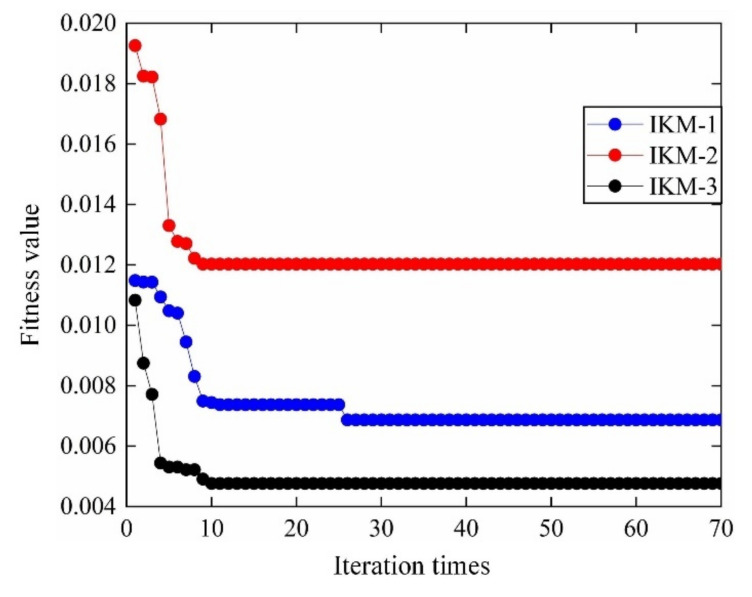
DMIGA optimization process of decomposed IKM.

**Figure 9:**
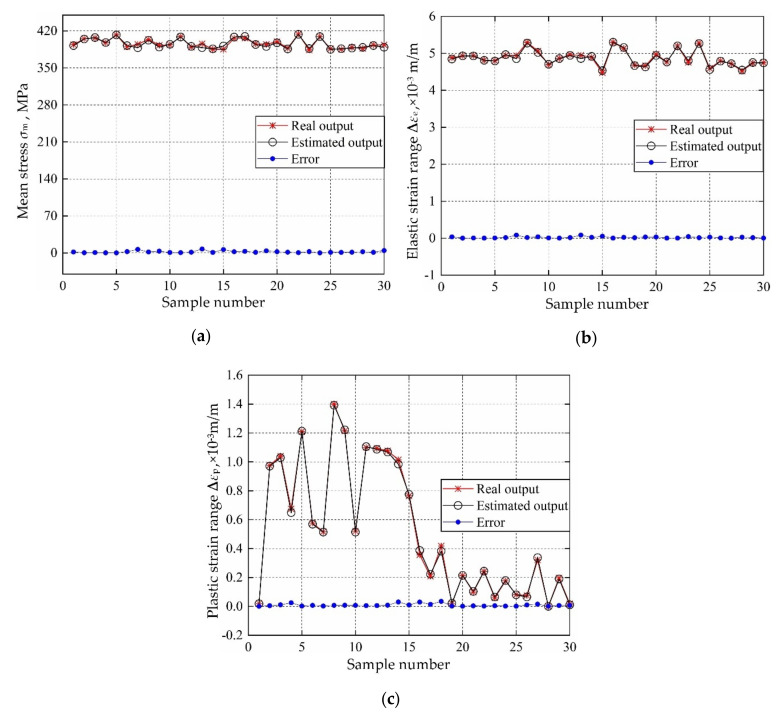
Prediction results of decomposed IKMs: (**a**) decomposed IKM-1, (**b**) decomposed IKM-2, (**c**) decomposed IKM-3.

According to the distribution characteristics of input variables in Table 1; Table 2, 107 sample data sets are extracted and be imported into FE simulation, to calculate the actual output responses (mean stress, elastic strain range, plastic strain range) sample data. The sample data sets are generated based on Latin Hypercube Sampling [45,46], which is promising to ensure that the generated sample covers the whole sampling domain. Then the 107 input variables & output responses are divided into training groups and test groups, where 30 for test groups and the remaining for training groups. Considering the training samples and decomposed IKM thought, the optimal decomposed IKMs for mean stress (decomposed IKM-1), elastic strain range (decomposed IKM-2) and plastic strain range (decomposed IKM-3) are constructed, the modeling process of decomposed IKMs with DMIGA is shown in Figure 8. From Figure 8, we discover that the stable optimization results can be obtained only through few iterations, which verifies the modeling efficiency of the proposed DMIGA. Moreover, by comparing real test outputs with the estimated outputs, the prediction effect of decomposed IKMs are obtained in Figure 9. Note that the data in Figure 9 comes from the testing dataset.

#### 4.3.2. Stress–Strain Prediction with Decomposed IKM Model

**Figure 10:**
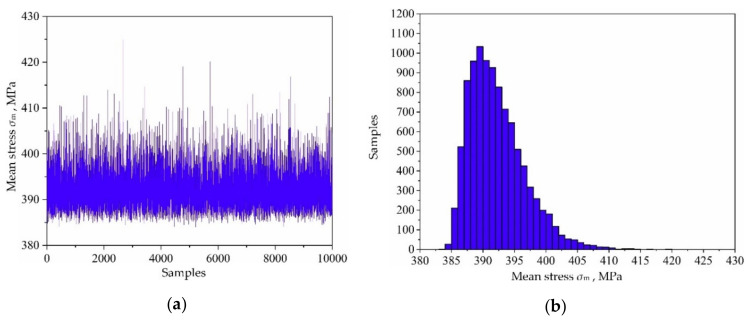
Output responses of decomposed IKM-1: (**a**) simulation history, (**b**) probabilistic distribution.

**Figure 11:**
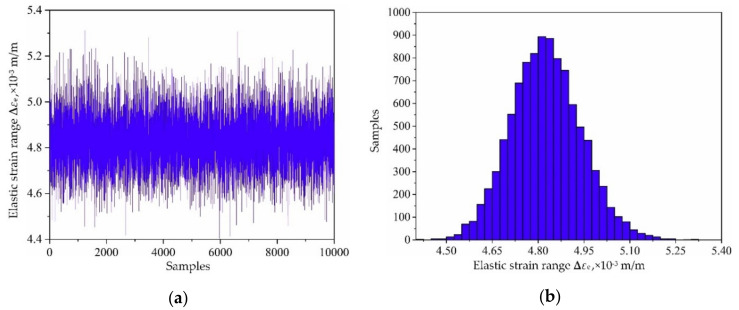
Output responses of decomposed IKM-2: (**a**) simulation history and, (**b**) probabilistic distribution.

**Figure 12:**
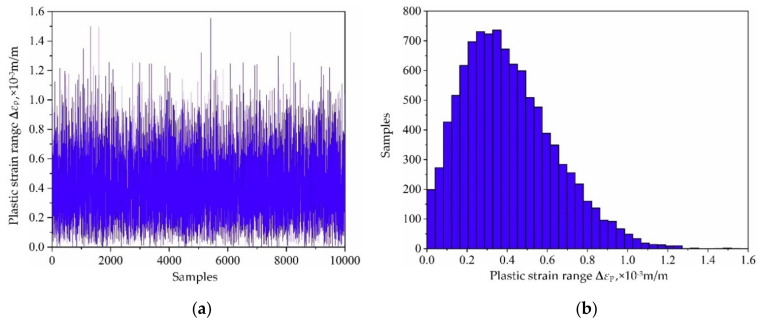
Output responses of decomposed IKM-3: (**a**) simulation history, (**b**) probabilistic distribution.

Based on the variable distribution features in Table 1 and the Latin hypercube sampling technique, 10,000 sets of input variables are extracted and be imported into the established decomposed IKM-1, decomposed IKM-2 and decomposed IKM-3, instead of the FE simulations. Then, the corresponding simulation history and probabilistic distribution of mean stress, elastic strain range and plastic strain range are obtained in Figure 10, Figure 11 and Figure 12. Owing to the influence of material plasticity, mean stress and plastic strain range approximately follow skewed distribution with mean values of 392.29 MPa and 4.13 × 10^−4^ m/m, standard deviations of 4.46 MPa and 1.64 × 10^−4^ m/m, respectively. The elastic strain range nearly obeys standard normal distribution with mean value 4.82 × 10^−3^ m/m and standard deviation 1.15 × 10^−4^ m/m.

#### 4.3.3. Sensitivity Analysis with Decomposed IKM Model

**Figure 13:**
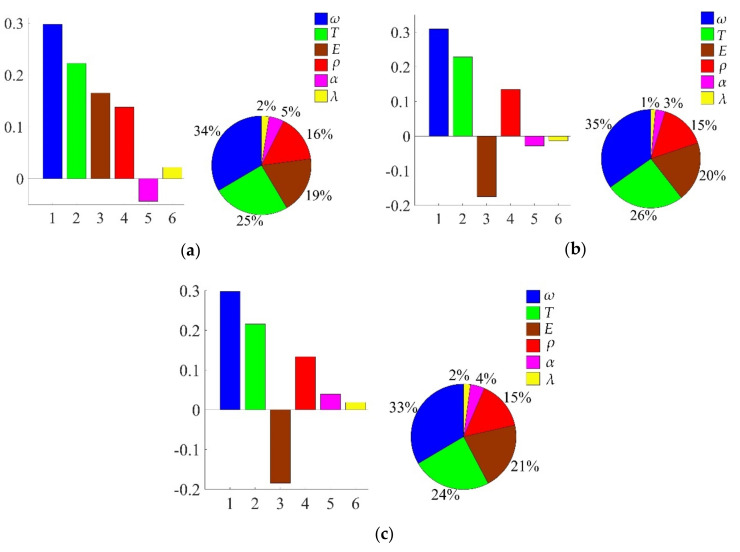
Sensitivities and effect probabilities with different decomposed output responses: (**a**) mean stress *σ*_m_, (**b**) elastic strain range **∆***ε*_e_, (**c**) plastic strain range ∆*ε*_p_.

**Figure 14:**
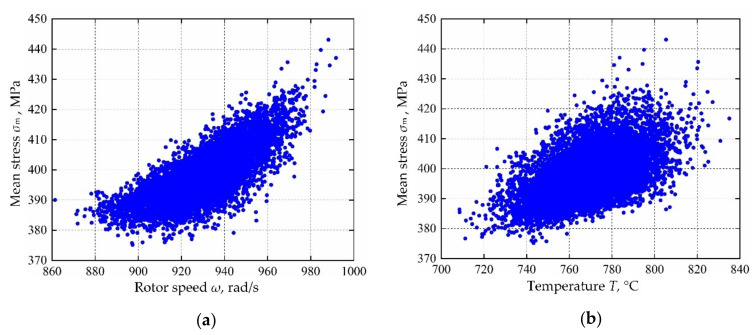
Scatter sketches of mean stress: (**a**) rotor speed, (**b**) temperature.

**Figure 15:**
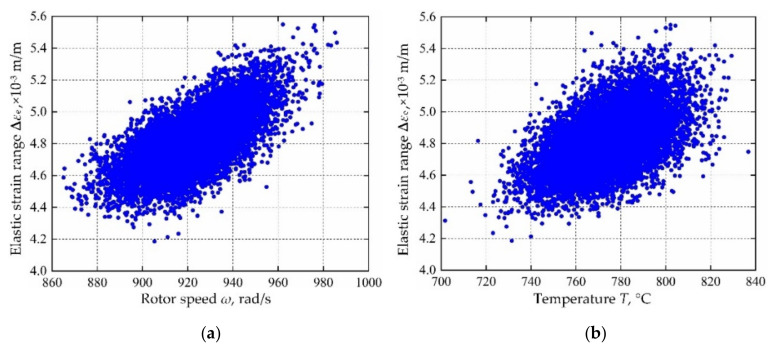
Scatter sketches of elastic strain range: (**a**) rotor speed, (**b**) temperature.

**Figure 16:**
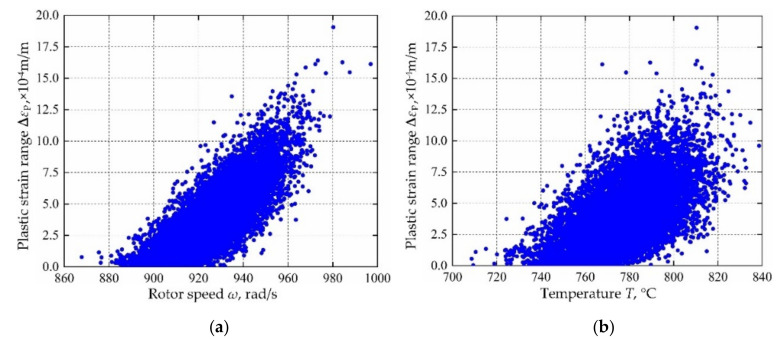
Scatter sketches of plastic strain range: (**a**) rotor speed, (**b**) temperature.

In view of the built decomposed IKMs, the sensitivity analysis is conducted based on Equation (22), sensitivities and effecting probability of input variables on decomposed output responses are obtained in Figure 13. Therein, the positive value of sensitivity indicates the positive change between output response and input variables, and vice versa. From Figure 13, we find that rotor speed *ω* and temperature *T* are the main affecting factors on mean stress *σ*_m_, elastic strain range **∆***ε*_e_ and plastic strain range **∆***ε*_p_. To further reflect the correlation of output response and prominent input invariables, the scatter sketches are drawn in Figure 14, Figure 15 and Figure 16, respectively. Note that the scatter points in Figure 16 are close to the X-axis because some extracted plastic strain range responses are close to zero.

### 4.4. Collaborative Fatigue Life Evaluation

#### 4.4.1. Collaborative IKM Modeling

**Figure 17:**
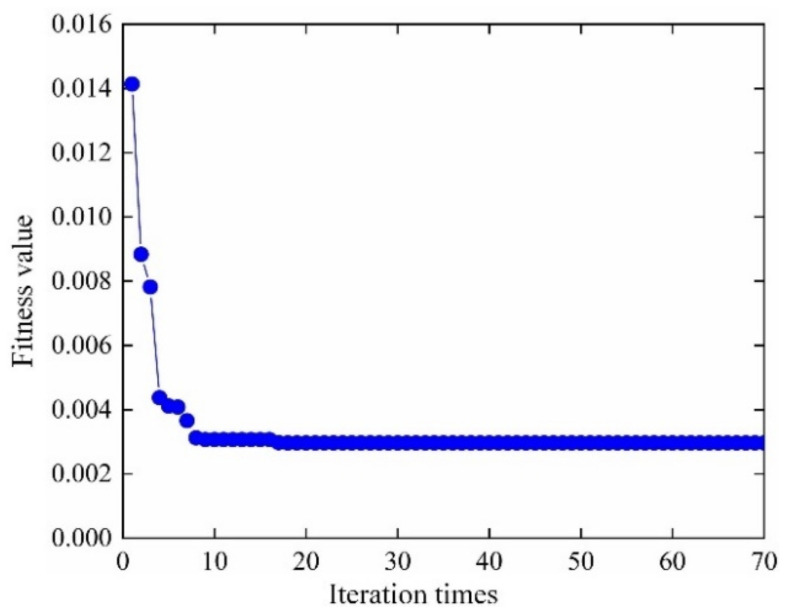
DMIGA optimization process of collaborative IKM.

**Figure 18:**
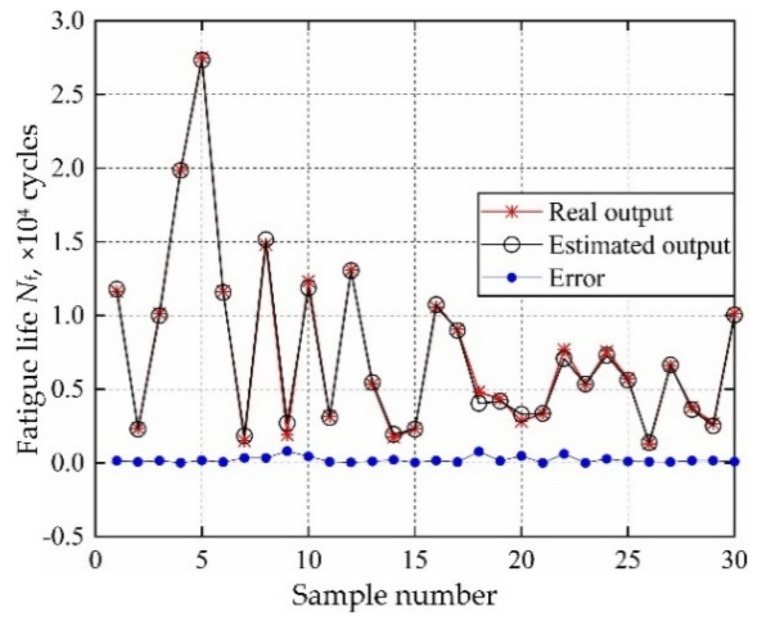
Prediction results of collaborative IKM.

The model parameters (fatigue strength exponent *b*, fatigue ductility exponent *c*, fatigue strength coefficient *σ*_f_′, fatigue ductility coefficient *ε*_f_′) and decomposed output responses (mean stress *σ*_m_, elastic strain range ∆*ε*_e_ plastic strain range ∆*ε*_p_) are taken as input variables of collaborative IKM, and the fatigue life of turbine rotor is taken as total output response. Similar to the establishment of decomposed IKM, 107 groups of samples are extracted, where 30 samples for testing samples and the remaining for training samples. The modeling process of collaborative IKM with DMIGA is shown in Figure 17. Through the comparison of real test outputs with the estimated outputs, the model performance of collaborative IKM is validated in Figure 18. Note that the data in Figure 18 comes from the testing dataset.

#### 4.4.2. Fatigue Life Evaluation with Collaborative IKM Model

**Figure 19:**
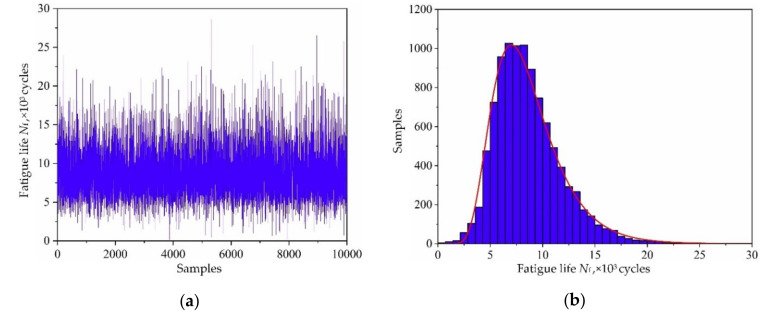
Fatigue life of turbine rotor: (**a**) simulation history, (**b**) probabilistic distribution.

Regarding the distribution features of four model parameters in Table 2 and the three decomposed output responses as input variables, the fatigue life *N*_f_ are obtained by simulating the collaborative IKM with 10,000 simulations. The simulation history and distribution feature of fatigue life *N*_f_ are shown in Figure 19. Obviously, the fatigue life *N*_f_ nearly obeys log-normal distribution. In view of the reliability analysis theory in Equation (24), the probabilistic fatigue life under reliability 99.87% is 3296 cycles. It should be noted that 3296 cycles are the probability fatigue life corresponding to 10,000 simulations, and the operating hours corresponding to 3296 cycles based on the flight load spectrum [32] are 1207 h.

#### 4.4.3. Sensitivity Analysis with Collaborative IKM Model

**Figure 20:**
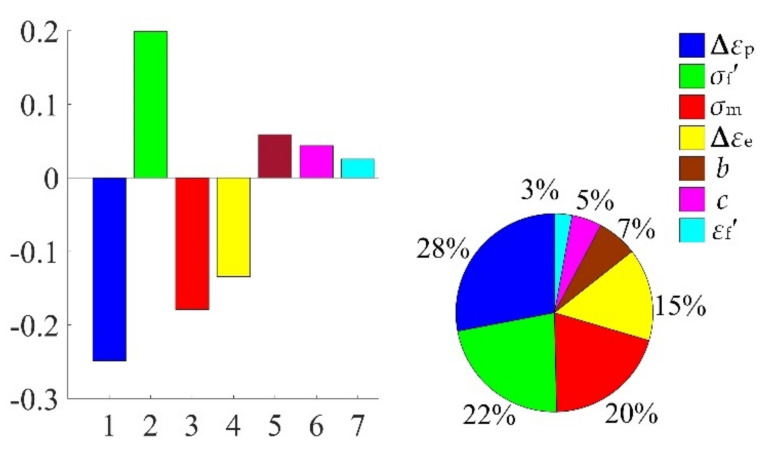
Sensitivities and effect probabilities of fatigue life.

**Figure 21:**
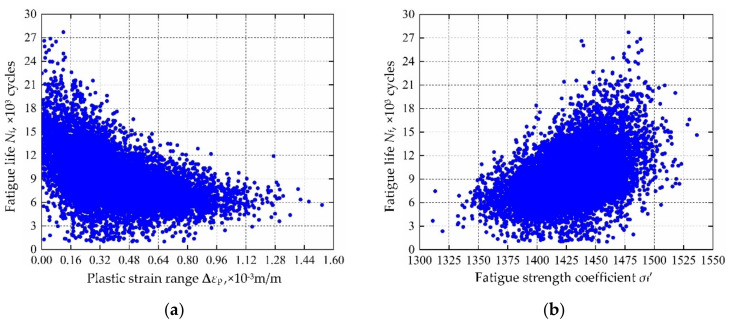
Scatter sketches of fatigue life: (**a**) plastic strain range, (**b**) fatigue strength coefficient.

Considering the collaborative IKM and the sensitivity analysis formula in Equation (25), the sensitivity analysis is accomplished, the sensitivities and influence probability of the input variables on fatigue life are obtained in Figure 20. The results show that plastic strain range ∆*ε*_p_ and fatigue strength coefficient *σ*_f_′ are the main factors affecting the fatigue life, accounting for 28% and 22%, respectively. Therefore, ∆*ε*_p_ and *σ*_f_′ should be preferentially regarded in fatigue reliability design of turbine rotor. Moreover, we also find that the decrease of ∆*ε*_p_ will result in the increase of fatigue life, while the increase of *σ*_f_′ will lead to the increase of fatigue life. The correlation between fatigue life and main influencing parameters are depicted in Figure 21. Note that the scatter points in Figure 21a are close to the Y-axis is owing to some plastic strain range responses are near to zero.

### 4.5. Method Validations

In this subsection, to verify the superiority of DCIKM, MCM, KM, IKM, DCRSM, and DCIKM are applied to perform the probabilistic fatigue life evaluation of turbine rotor, respectively. During the evaluation, MCM, KM and IKM calculate the relationship between input variable *X*′ = [*ω*, *T*, *ρ*, *E*, *λ*, *α*, *σ*_f_′, *c*, *b*, *ε*_f_′] and output response *N*_f_ directly, while DCRSM and DCIKM adopt parallel calculation by simulating elastic strain range Δ*ε*_e_, plastic strain range Δ*ε*_p_, mean stress *σ*_m_, and fatigue life *N*_f_ in several computer devices. The computing costs and computing precision of different evaluation methods are compared in Table 4 and Table 5, respectively.

In terms of computing efficiency, it can be seen from Table 4 that the computational costs of DCIKM is lower than that of traditional KM, IKM and DCRSM, while the computational costs of traditional KM, IKM, DCRSM and DCIKM are far lower than MCM. The efficiency superiority of DCIKM is because: (i) for complex objective function with multi-modality characteristics, the DMIGA is conductive to find optimal Kriging parameters efficiently; (ii) owing to the decomposed collaborative strategy employed, the DCIKM only needs to process small quantity of input variables in modeling, which saves the simulation time significantly. Therefore, the proposed DCIKM is promising to improve computing efficiency in probabilistic fatigue life evaluation. 

In terms of computing accuracy, as shown in Figure 8; Figure 18, the prediction results are basically close to the real outputs. From Table 5, we find that the proposed DCIKM holds the higher prediction accuracy than the traditional KM, IKM and DCRSM, and is close to the direct MCM. The main reasons are: (i) the global optimum θ* is searched and the optimization accuracy is guaranteed by using the developed DMIGA, which effectively enhances the modeling accuracy of Kriging model; (ii) For the multi-layer multi-response probabilistic evaluation, the decomposed collaborative strategy reduces the nonlinear degree of surrogate modeling and thereby improves the model accuracy. Therefore, the proposed DCIKM holds high computing accuracy in probabilistic fatigue life evaluation.

## 5. Conclusions and Outlooks

To improve the simulation efficiency and accuracy for probabilistic fatigue life evaluation of turbine rotor, a decomposed collaborative intelligent Kriging modeling (DCIKM) approach is presented in this study. To guarantee the model efficacy, the DMIGA intelligent algorithm is introduced to find the optimal Kriging parameter and build accurate intelligent Kriging model (IKM), the decomposed collaborative strategy is employed to further reduce the nonlinearity of IKM. The effectiveness of the proposed DCIKM approach is verified by the probabilistic fatigue life assessment of turbine rotor. Some conclusions are summarized as follows:

1. The simulation history and distribution characteristics of fatigue life are obtained and the reliability-based fatigue life *N*_f_ = 3296 cycles is recommended for the turbine rotor fatigue life design, which is conducive to greatly enhance the safety performance of turbine rotor.
2. The sensitivity analysis results show that rotor speed and gas temperature are the main factors on mean stress, elastic strain range and plastic strain range, while plastic strain range and fatigue strength coefficient are the major factors on fatigue life, which provides a valuable guidance for further optimization of turbine rotor.
3. Methods comparison (MCM, KM, IKM, and DCRSM, DCIKM) illustrates that the proposed DCIKM holds superiority in computing efficiency and accuracy. Accordingly, it is proved that the intelligent algorithm searching for optimal Kriging parameters is promising to build a higher-precision Kriging model. Moreover, the decomposed collaborative strategy is suitable to decrease the nonlinearity of probabilistic design of turbine rotor.

Although this study provides a feasible and efficient approach for probabilistic fatigue life evaluation of turbine rotor, limitations exist. Most deviations from expected responses are attributed to incomplete factors considered in this study. Moreover, the trained surrogate model in this paper is only available for the specific turbine rotor structures and cannot be directly applied to other turbine rotor structures. Furthermore, for other more complex large-scale fatigue life evaluation problems, the method’s performance should be further investigated in future work.

## Figures and Tables

**Table 1:** Distribution characteristics of physical variables.

Random Variables	Mean	Standard Deviation	Distribution
Rotate speed *ω*, rad/s	922	18.4	Normal
Gas temperature *T*, k	773.2	15.5	Normal
Density *ρ*, 10^−9^ t/mm^3^	8.21	0.164	Normal
Modulus of elasticity *E*, GPa	163	3.26	Normal
Heat conductivity *λ*, W/(m °C)	21.4	0.428	Normal
Thermal expansion coefficient *α*, 10^−6^ °C	13.8	0.276	Normal

**Table 2:** Distribution characteristics of model variables.

Random Variables	Mean	Standard Deviation	Distribution
Fatigue strength index *b*	−0.1	0.002	Normal
Fatigue ductility index *c*	−0.84	0.0168	Normal
Fatigue strength coefficient *σ*_f_′	1419	28.38	Normal
Fatigue ductility coefficient *ε*_f_′	0.505	0.0101	Lognormal

**Table 3:** Variation characteristics of three nonlinear parameters.

Temperature (°C)	100	200	300	400	500	600	700	800	900
*E*, GPa	205	196	182	173	163	163	159	141	134
*λ*, W/m °C	12.1	14.2	16.7	18.8	21.4	23.7	26.2	27.6	28.9
*α*, 10^−6^ °C	11.6	12.3	12.4	13.3	13.8	14.4	15.1	15.7	16.5

**Table 4:** Comparison of computing time for reliability analyses of five methods.

*n*	MCM	KM	IKM		DCRSM		DCIKM	
	Time, s	Time, s	Time, s	Improved Efficiency, %	Time, s	Improved Efficiency, %	Time, s	Improved Efficiency, %
10^2^	5754	45.7	40.1	12.25	31.9	30.19	22.7	50.33
10^3^	60,890	47.1	41.2	12.53	32.8	30.36	23.2	50.74
10^4^	798,954	49.8	43.1	13.65	34.6	30.52	24.5	50.80
10^5^	—	58.7	50.4	14.14	39.7	32.37	28.3	51.79

Note that the improved efficiency is calculated by: Improved efficiency = (*T*_c_ − *T*_KM_)/*T*_KM_, where *T*_c_ is the calculation time of the compared method, *T*_KM_ the calculation time of KM method.

**Table 5:** Reliability analysis results of five methods for turbine rotor fatigue life (*N* = 3296 cycles).

*n*	MCM	KM		IKM		DCRSM		DCIKM	
	Reliability	Reliability	Precision, %	Reliability	Precision, %	Reliability	Precision, %	Reliability	Precision, %
10^2^	0.92	0.81	88.04	0.90	97.83	0.87	94.57	0.91	98.91
10^3^	0.984	0.915	92.99	0.971	98.68	0.942	95.73	0.975	99.09
10^4^	0.9977	0.9521	95.43	0.9969	99.92	0.9731	97.53	0.9972	99.95
10^5^	—	0.9579	96.01	0.9971	99.94	0.9739	97.61	0.9970	99.93
